# Supplementary material for: Cannabigerol Alleviates Liver Damage in Metabolic Dysfunction-Associated Steatohepatitis Female Mice via Inhibition of Transforming Growth Factor Beta 1
Source: Nutrients. 2025 Apr 30;17(9):1524. doi: 10.3390/nu17091524 (PMC12073672; doi:10.3390/nu17091524)
Supplement: Supplementary file 1 [file nutrients-17-01524-s001.zip › nutrients-3537032-supplementary.pdf]

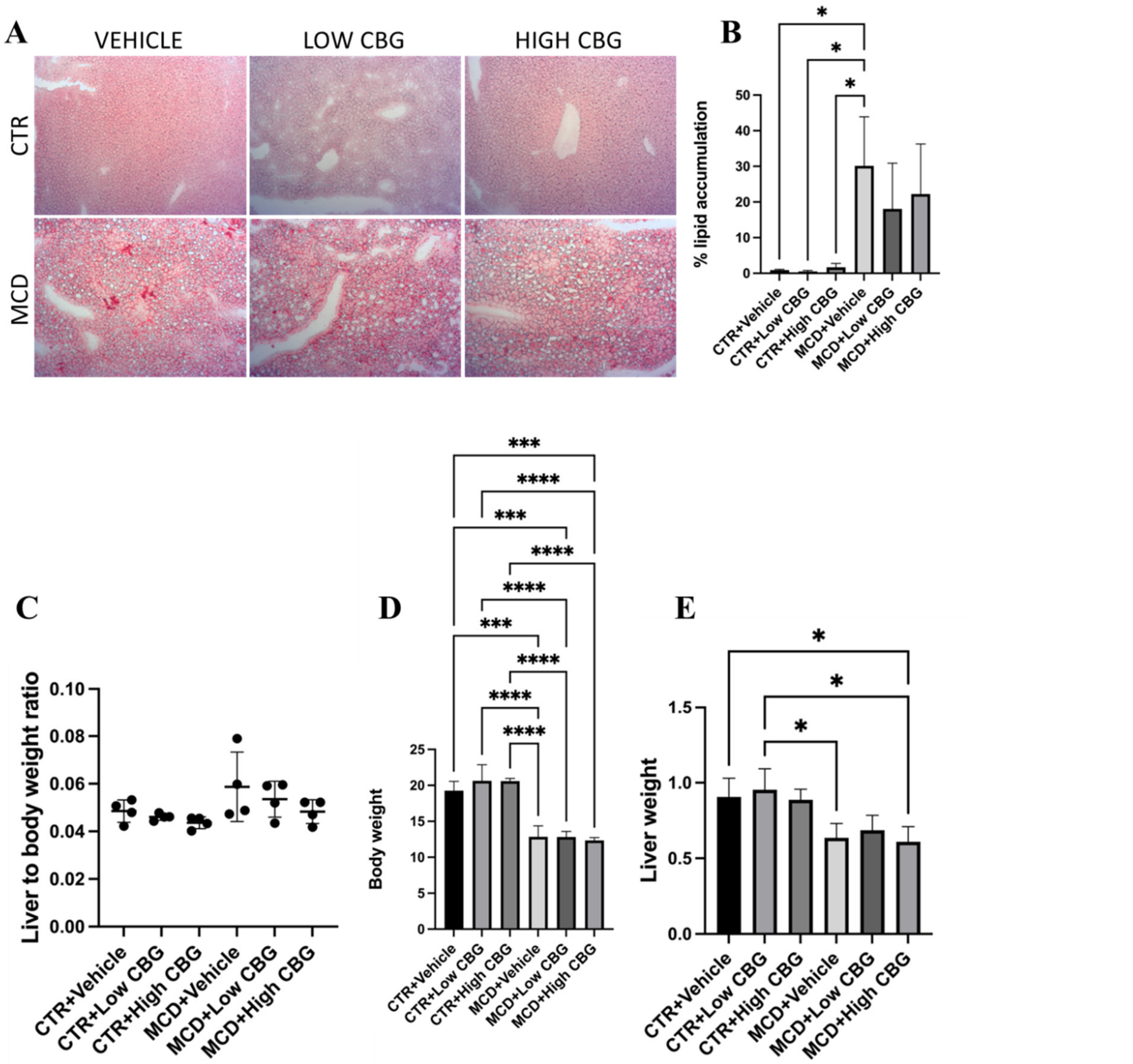

Supplementary Figure S1: Evaluation of hepatic steatosis and overall liver health. (A) Oil red O staining was done to stain accumulated lipid or fat in frozen liver section. Red colors represent the lipid in liver tissues. Original magnification, × 100. (B) Quantification of the level of lipid accumulation. Quantification was done by ImageJ software. (C) Graph of individual values from liver to body ratio of mice (p value =0.1622). (D, E) Measurement of body weight and liver weight of mice of different experimental groups. ∗P <0.05; ∗∗p <0.01; ∗∗∗p < 0.001; ∗∗∗∗p < 0.0001.

Supplementary Table S1. *List of Antibodies and Anti-mouse Secondary Antibody Fluorescent Dyes.*

Antibody/Fluorescent dye	Company	Dilution	Catalogue	Source
**FcεRI**	Biolegend	1:100	134310/13409	Armenian Hamster
**CD45**	Life tech	1:25	14-0451-82	Rat
**CK19**	DHSB	1:25	TROMA-III	Rat
**TGFb1**	life technology	1:100	21898-1-AP (PA1-29032)	Rabbit
**8-OHdG**	Santa Cruz	1:100	SC-66036	Mouse
**Desmin**	Novus Biological	1:20	AF3844	Goat
**Tryptase (Tpsb2)**	LSBio	1:20	LS-B15255	Mouse
**CD3**	Biolegend	1:200	100235	Rat
**CD4**	Biolegend	1:400	100509	Rat
**CD8a**	Biolegend	1:200	100751	Rat
**CD115**	Biolegend	1:400	135509	Rat
**CD11b**	Biolegend	1:400	101205	Rat
**Ly6C**	Biolegend	1:400	128007	Rat
**Alexa Fluor® 647**	Abcam	1:1000	ab150155	Donkey
**Alexa Fluor® 594**	Abcam	1:1000	405306	Goat
**Alexa Fluor® 488**	Abcam	1:1000	AB150073	Donkey
**Alexa Fluor® 555**	Abcam	1:1000	ab150130	Donkey
